# Preliminary Research on the Sustainable Determinants of Taiwanese Ecotourism with the International Standards

**DOI:** 10.3390/ijerph192114489

**Published:** 2022-11-04

**Authors:** Chih-Cheng Huang, Yung-Kuan Chan, Ming Yuan Hsieh

**Affiliations:** 1 Department of Leisure & Recreation, National Formosa University, Yunlin 403632, Taiwan; 2 Department of Management Information Science, National Chung Hsing University, Taichung 402204, Taiwan; 3 Department of International Business, National Taichung University of Education, Taichung 403454, Taiwan

**Keywords:** Taiwanese ecotourism, sustainable ecotourism international standards, social learning theory (SLT), quantitative and qualitative analyses

## Abstract

To analyze Taiwanese ecotourism with international standards, this research employed the social learning theory (SLT) to identify the determinants of Taiwanese community ecotourism with the international standards. This basic theory of social psychology comprehensively assays the interplays and interconnections among the three analytical perspectives (ecotourism tours, destinations, and accommodations), the four essential issues (management, social, cultural, and environment) of the Global Sustainable Tourism Council, the six assessed dimensions of ecotourism resolution from the WCC, and the twenty-nine assessed indexes of the KES ecotourism evaluations. It was then possible to comprehensively explore the sustainable determinants of Taiwanese ecotourism with the international standards through the FA of quantitative and qualitative analyses in combination with the highest research validity, reliability, representativeness, and accuracy. After analyzing the evaluated measurements, the empirical and valuable conclusions and findings are (1) these analytical perspectives, appraised attitudes, evaluated criteria, and sub-criteria positively advance Taiwanese ecotourism with the international standards (PITEEICIS); (2) the sustainable determinants of Taiwanese ecotourism with the international standards include the Support for Capacity Building of the Local Community (SCBLC), Cooperation with the Local Community (CLC), Supports for the Local Enterprises (SLE), Local Participation and Benefits Sharing Duties (LPBSD), Tourist Management (TM), and Responsible Tourist Behaviors Inducement (RTBI). Importantly, the majority of ecotourism industrialists and experts still focus on the economic benefits, such as supporting the local community and enterprises, rather than tourist behavior inducement to stimulate ecotourism participation in order to promote and advance the Taiwanese ecotourism to the international standards; (3) in order to promote Taiwanese ecotourism to the international conventions, the Taiwanese government and organizations in ecotourism should contribute toward the local welfare and create and design various training programs and courses to enhance local community’s awareness and capability of ecotourism development in order to establish a complete system that stimulates the ongoing planning and decision making of local community participation and regularly monitors, records, and reflects their opinions, based on the area’s history, culture, and natural attributes, to develop and sell sustainable local products by creating fair trade principles and valuable products.

## 1. Introduction

With the rapid increase in global Gross Domestic Product (GDP) and swift development of transportation technology, domestic and international tours have increasingly become entertainment for contemporary people. The majority of people would like to discover novel news, information, and life experiences through a series of tours. In addition, many local regions, areas, and communities also utilize tourism to expose their news, information, and culture to the public in order to attract more people to visit, which then develops the regional economy and reputation. In the wake of the booming tourism development, each ecological environment has confronted a bulk of unpreceded and irreversible destruction and hyper-pollutions [1]. In order to effectively and efficiently diminish and minimize the shock of various destructions and hyper-pollutions from tourism, ref. [2] has consolidated the expert ecological ideas into vocational principles to create ecotourism. Importantly, not every economic tourism activity destroys the environmental condition. By recognizing many ecological catastrophes in concrete species extinctions (such as indigenous species extinction) and abstract capital eliminations (such as aboriginal culture elimination), numerous national governments, local communities, and public residents have paid more attention to the economic development benefits with consideration to the diminishment of various environmental resources and hyper-pollutions. The empirical practice of ecotourism was implemented in lobbying for the environmental protection of phoenicopterus ruber on the northern wetlands of the Yucatán while the development of wharf construction was pursued; ref. [3] advocated the ecological conservation of phoenicopterus ruber to attract more birdwatching tourists to perk up the local economy without destroying the local ecological environment. Originally, the beginning of the ecotourism concept focused on the balance between economic development and ecological protection in order to decrease the various impacts of tourism development on the ecological environment.

Through the higher transmission speed of wireless technology, the sources used to surf and download local information, such as local news, local real-time video, local transportation information, local on-time announcements, local festival schedules, etc., can also be used by local organizations to expose the local news, messages, and statements in real-time without time and space restrictions. Furthermore, people now would like to easily visit anywhere in the world, which depends on the swift development of transportation technology and convenient infrastructure [4]. Therefore, according to the 2015 annual report of the World Conservation Congress (WCC), the global tourism industrial output value reached 1.2 trillion United States Dollars (USD) in 2014 and increased to roughly 4 trillion USD in 2018, accounting for 2 out of 11 jobs overall in the world. However, the global tourism industrial output value dropped to 800 billion USD in 2021 due to the 2019 global epidemic impact of the Coronavirus Disease-2019 (COVID-1), indicating that tourism plays a critical role in contemporary society due to the rapid development of wireless and transportation technologies [5]. Nature-based tourism is a major tourism sector, comprising more than 25% of the global travel market; however, tourism industrialists have overlapping and differing definitions and interpretations as well as few precise standards for ecotourism, nature-based tourism, or geo-tourism (based on geodiversity and geological heritage without the consideration of ecotourism). When examining the Taiwanese domestic tourism market, the industrial output value was still approaching approximately 100 billion USD under the severe shock of COVID-19, according to the 2021 annual report of the Tourism Bureau in Taiwan.

In the book, *Tourism, ecotourism, and protected areas*, by the International Union for Conservation of Nature (IUCN) International Union for Conservation of Nature (IUCN), ecotourism is defined as “the environmentally responsible travel and visitation to relatively undisturbed natural areas, in order to enjoy and appreciate nature (and any accompanying cultural features—both past and present) that promotes conservation, has low visitor impact, and provides for beneficially active socio-economic involvement of local populations” [6]. “As the tour has become the mainstream activity in the contemporary society, the sustainability has also become the mainstream issue in the modern tourism because the more tourists are directly and obviously to bring in the more tourism output value” [6].

For this reason, the WCC of IUCU has unanimously passed a resolution on the international standards for sustainable ecotourism in association with the 17 Sustainable Development Goals (SDGs) of the 2030 agenda for the sustainable development of the United Nations (UN) Department of Economic and Social Affairs. As for the Eco-friendly Development (ED) in ecotourism resolution from the WCC, the eleventh (sustainable cities and communities with decent work and economic growth), twelfth (responsible consumption and production), and fourteenth (life below water) SDGs are the measures recommended for sustainable economic growth, consumption, and production practices and the conserved and sustainable use of nature and natural heritage. In detail, the eleventh SDG aims to expedite, maintain, contain, and increase economic sustainability in order to advance the establishment of safe residential sections with disaster prevention for each individual. The twelfth SDG was created to ensure sustainably consumed and productive models and the fourteenth SDG was promoted for the sustainable development of ocean and water natural resources. In regards to the Responsible Management (RM) ecotourism resolution from the WCC, the fifth (gender equality), eighth (decent work and economic growth), ninth (industry, innovation, and infrastructure), and seventeenth (partnerships for the goals) SDGs are effective approaches for ecotourism organizations. The ecotourism organizations are supposed to have the responsibility of advancing gender equality in each employee to facilitate the efficient production of sustainable economic growth in order to expand the sustainable global collaboration relationship. Importantly, organizations in ecotourism should further establish safe, tolerant, innovative industrialization with disaster prevention [7].

In terms of the Environmental Conservation (EC) in ecotourism resolution from the WCC, the thirteenth (climate action) and fifteenth (life on land) SDGs are effective goals for ecotourism organizations. Ecotourism organizations have to take aggressive actions to confront and diminish the impact of global climate change through a series of actions such as sustainable terrestrial ecosystem utilization and the sustainable management of forests as well as the avoidance of desertification, soil degeneration, and loss of biodiversity. With respect to the Genuine Ecotourism Experiences Provision (GEEP) in ecotourism resolution from the WCC, the fourth (quality education) and sixteenth (peace, justice, and strong institutions) SDGs establish the effective magnanimity of ecotourism organizations. Ecotourism organizations have to provide empirical experiences and provisional tour activities and programs to advance inclusive and equal quality education to establish the environmental conservation lifelong learning mechanism in order to strengthen an inclusive, equal, and sustainable society [8]. In regard to the Responsible Tourist Behaviors Inducement (RTBI) in ecotourism resolution from the WCC, the sixth (clean water and sanitation) and seventh (affordable and clean energy) SDGs provide effective approaches for ecotourism organizations.

Ecotourism organizations are necessary to conduct the tourists in such a way as to maintain and protect the natural water, resources, energies, and sanitation structure throughout tourism activities. Ultimately, in reference to the Local Participation and Benefits Sharing (LPBS) ecotourism resolution from the WCC, the first (no poverty), second (zero hunger), third (good health and well-being), and tenth (reduced inequalities) SDGs indicate effective structures for ecotourism organizations. Ecotourism organizations have to plan for the diminishment of poverty through the accomplishment of food security and improvement of nutritional status in order to achieve good public health and well-being. Hence, ecotourism not only focuses on the expedition of environmental protection but also emphasizes cultural conservation as well as benefits the local economy by the presence of tourists to continuously achieve the sustainable triple-bottom balance between the earth’s environment, social humanities, and economic benefits [9].

Therefore, the World Tourism Organization (UNWTO) and United Nations Statistics Division (UNSD) have started to collect exhaustive ecotourism data to understand the complete performance and development of global ecotourism to construct the evaluated international standards and evaluated indicators of development of the suitability of global tourism from the economic, environmental, and social dimensions of the sustainable tourism information diagram in 2017, the year of international sustainable ecotourism [10]. Subsequently, the UNWTO has devoted itself to developing a series of evaluation indicators through existing economic, environmental, and social international standards for instituting an easy and convenient evaluation framework to discuss, manage, and integrate the massive tourism-related data and information. This massive amount of information and data is able to provide and produce enough useful information for numerous tourism stakeholders, such as tourists, tourism industrialists, community residents and staff, tourism governments, etc. to administer and make suitable decisions for the empirical demands of residents in the sustainable development of global ecotourism [11,12].

Subsequently, the WCC has officially announced the One Program to request the director general, commissions, and members to support an initiative for the international standards in ecotourism at its session in Hawai‘i, United States of America, 1–10 September 2016 that (1) practically expands the sustainable tourism guidelines to cover the best ecotourism practices, including an updated IUCN definition of ecotourism, relevant standards and indicators for culturally sensitive community engagement and welfare, environmental learning, and appropriate infrastructure and tourist behaviors, to seek the prevention of a negative anthropogenic influence on species and ecosystems, (2) empirically implements, in existing national, regional, and international certification schemes, standards and guidelines focused on ecotourism in order to improve and promote conservation outcomes by encouraging the uptake of the best practices and adherence to and strengthening of globally accepted international standards, and (3) systemically designing, creating, and delivering a series of training opportunities for ecotourism governance, auditing, and certification as well as the implementation of the best practices for ecotourism development and management.

In regard to the development of Taiwanese ecotourism, the Tourism Bureau of the Executive Yuan officially established the Taiwan Ecotourism Association (TEA) in 2001 by organizing the government, civil powers, and resources to facilitate the ecotourism development in Taiwan with the aim of promoting the sustainable and wise use of Taiwan’s natural, cultural, and ecological resources in ecotourism. Based on the fundamental goals of the TEA, there are five main purposes: (1) conducting training courses and workshops covering multiple aspects of ecotourism; (2) promoting ecotourism by partnering with industries; (3) setting up a platform to consolidate efforts from all parties for ecotourism development, (4) establishing an evaluation and certification program to enhance the quality of ecotourism, and (5) reinforcing international cooperation and coordinating demands from international markets. In order to empirically elaborate on tourism operations, community development, biodiversity, geodiversity, geological heritage, places of geological interest, wildlife, and the natural environment, Taiwanese ecotourism must explore its sustainable determinants with the international standards.

However, the driving business contents and organization framework, the critical demand for contemporary Taiwanese ecotourism, and the evaluation indicators and certificates with international standards are the current severe shortages in the TEA. Therefore, after making a comprehensive survey of the research on ecotourism development in Taiwan, there was no one official study able to focus on detecting the sustainable determinants of Taiwanese ecotourism with international standards. In order to realistically provide the research evaluations and assessments of the sustainable determinants of Taiwanese ecotourism with the international standards of the Taiwanese government, parastatal ecotourism organizations and associations and ecotourism researchers, developers, and tourism industry professionals, as well as the national, regional, and international certification schemes, standards, and guidelines on this research topic were used to concentrate on ecotourism to encourage the adoption of international standards and norms with the updated IUCN definition and guidelines to be analyzed in-depth and discussed.

**Figure 1:**
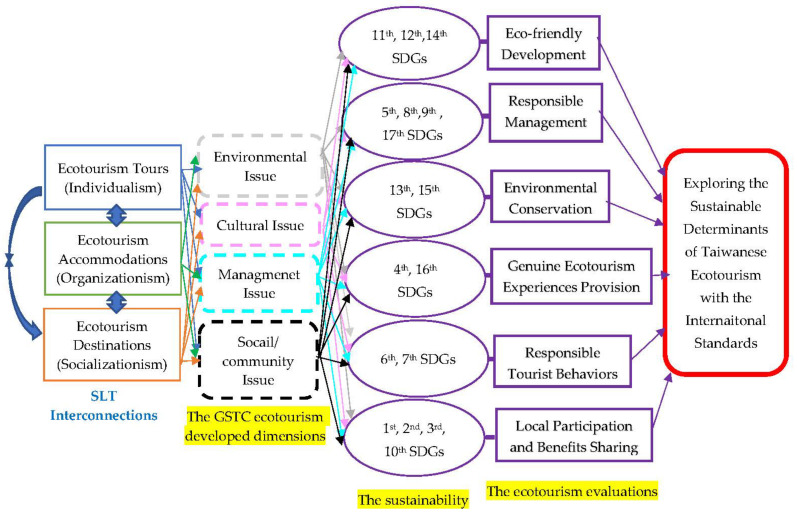
Main analytical concept and hierarchy.

For this reason, these sustainable determinants are able to promote and seek auditing and certification for Taiwanese ecotourism in landscapes and seascapes of conservation value, according to international standards. In order to empirically explore the sustainable determinants of ecotourism with the international standards, the ecotourism destinations, accommodations, and tours have been defined as the analytical perspectives because the tours focus on the individual’s feeling, accommodations depend on the organizations in ecotourism, and the destinations assemble the local natural and cultural resources and assets. Then, the four creditable dimensions (management, social/community, cultural, and environmental issues) of the GSTC were classified as the appraised attitudes. Lastly, the six assessed dimensions of ecotourism resolution from the WCC, in association with the seventeen SDGs of the 2030 agenda for sustainable development and the twenty-nine assessed indexes of the KES ecotourism evaluations in connection with the six assessed dimensions of the ecotourism resolution from the WCC, were extensively discussed and considered as the evaluated criteria and sub-criteria in this research. Therefore, this research has innovatively employed the social learning theory (SLT) [13] to form the main analytical hierarchy and concept of the three analytical perspectives (Ecotourism Tours, ET; Ecotourism Destinations, ED; and Ecotourism Accommodations, EA) in order to comprehensively appraise the interplays and dependences among ecotourism tours, destinations, and accommodation. The SLT was able to examine the correlations among the four essential issues (Management Issue, MI; Social Issue, SI; Cultural Issue, CI; and Environmental Issue, EI) of the GSTC, the six assessed dimensions of ecotourism resolution from the WCC, and the twenty-nine assessed indexes of the KES ecotourism evaluations in the main analytical hierarchy and concept, as reflected in Figure 1.

The core reason the SLT was created was to extensively discuss the dependences and influences among individual behaviors, organizational operation, and performance as well as social development and tendency [14,15]. The individual behaviors of ecotourism tours were directly able to form the ecotourism accommodations of organizational operation and performance and reversely, the ecotourism accommodations of organizational operation and performance were also able to indirectly influence the ecotourism tours of individual behaviors because each individual is the basic unit of the organization [16,17]. In addition, the ecotourism accommodations of organizational operation and performance were directly instituted by the ecotourism destinations of social development and tendency, and conversely, the ecotourism destinations of social development were further able to dependently facilitate the ecotourism accommodations of organizational operation and performance [18,19]. Ultimately, the ecotourism destinations of social development and tendency immediately dominated the individual behaviors, and oppositely, the ecotourism tours of individual behaviors were able to potentially affect the social development and tendency because society is gathered and assembled from each individual as reflected in Figure 1.

In statistical measurements, the Factor Analysis (FA) of quantitative analysis [20] was applied to administer large-scale questionnaires for inducing the appraised commonalities among each attitude, criterion, and sub-criterion in the main analytical hierarchy of the SLT in order to achieve the highest validity and representativeness because the FA of quantitative analysis was created to identify and induce the critical determinants through large-scale questionnaires in the social research science fields. Importantly, the Analytical Network Process (ANP) [21] of qualitative analysis was further employed to conduct the professional expert’s weighted questionnaires by measuring the assessments among each attitude, criterion, and sub-criterion in the main analytical hierarchy of the SLT in order to achieve the highest reliability and accuracy because the ANP was initially induced to systematically and hierarchically establish the evaluated model to testify the interconnections among these determinants.

## 2. Methodological Literature

### 2.1. Literature on the Ecoturism International Standard Certification

Currently, over two hundred green certificates and certifications in the world have been created to offer to the various tourism-related industries and companies to apply based on each of their specific demands which have instituted their ecological certificate recognition and mechanism. These green and ecological certificate recognitions are able to assist tourism-related industries to concretely strengthen their comparison and sustainability in regard to their operational costs and corporate images in order to contribute to environmental protection and cultural preservation. Focusing on the evaluated indicators and certificate and ecotourism development, the Korea Tourism Organization (KTO) of the Korean Ministry of Culture and Tourism (KMCT) has instituted the Korean Ecotourism Standards (KES) since 2010 furthermore, the KES has been proofed and recognized by the global ecotourism certificate system of the Global Sustainable Tourism Council (GSTC), which means any tourism organizations can obtain the accreditations of the KES and CSTC if they can respect regulations and pass the KES ecotourism evaluations. In detail, the destinations (socializationism), accommodations (organizationism), and tours (individualism) have been identified as the three core perspectives from the KES ecotourism evaluations [22]. Furthermore, the EFD, RM, EC, GEEP, RTBI, and LPBS in ecotourism resolution from the WCC were classified as the evaluated criteria to estimate the three core appraised perspectives from the KES ecotourism evaluations.

First of all, there are two assessed sub-criteria in the EFD evaluated criteria [23] which include 1. Resources Protection (RP): the tourism organizations are supposed to have policies and systems to evaluate, rehabilitate, and conserve natural and cultural resources including scenic views for conserving creatures, habitats, species, and ecosystems [24]; 2. Eco-friendly and Cultural-friendly Buildings and Infrastructures (ECBI): the tourism organizations have to possess the ecological strategies for the planning, siting, design, construction, renovation, operation, and demolition of buildings and infrastructures that have been adopted and implement them to contribute to the sustainability, identity, and resilience of the area; especially, where appropriate, sites and facilities have applied a universal design [25].

Secondly, there are eleven sub-criteria in the RM evaluated criteria and which are 1. Compliance with Laws (CL): the tourism organizations have to respect national and international laws associated with ecotourism development, management, and regulations covering public health, safety, labor, cultural, and environmental aspects. 2. Financial Insurance (FI): the tourism organizations have to plan and implement insurance contracts to protect tourist destinations, residents, facilities, and visitors. 3. Sustainable Management Strategies (SMS): the tourism organizations have to establish and administer multi-year (destination) management strategies. These strategies cover environmental, economic, social, cultural, quality, health, safety, and aesthetic issues in order to publicly develop and increase public participation and the appropriate financial funds and subsidies. 4. Management Units (MU): the tourism organizations must have effective staff and individual social responsibility for a coordinated approach to reach sustainable ecotourism. The effective staff is supposed to be appropriate for the ecological conservation scope and deal with environmental, economic, social, cultural, quality, health, and safety issues [26].

They establish an efficient system for equal employment, equal training, fair wages, fair evaluation, and periodic guidance and training (including advanced levels) regarding staff roles and responsibilities for sustainable ecotourism in collaboration with the stakeholders in the ecotourism system. 5. Monitoring (M): the ecotourism organizations have to establish a periodically reviewed, improved, and monitored proclamation system to monitor, publicly report, and respond to environmental, economic, social, cultural, tourism, and human rights issues, including both direct and indirect ecotourism impacts annually, at least [27]. 6. Strategy for Climate Change (SCC): the ecotourism organizations have to establish a climate change recording and evaluation system to identify the risks and opportunities associated with environmental change to set up strategies for the development, siting, design, and management of facilities in order to advance the public education on climate change for residents, visitors, and tourists [28]. 7. Planning Regulations (PR): the ecotourism organizations have to provide planning guidelines, regulations, and/or policies regarding requiring an environmental, economic, and social impact assessment and integrating sustainable management and development, contained land usage, design, construction, and demolition. 8. Crisis and Emergency Management (CEM): the ecotourism organizations need to have a series of crisis and emergency response plans and regulations that are updated on regular bases and these plans have to be regularly practiced and trained by the staff, residents, tourists, and visitors [29]. 9. Visitor Satisfaction (VS): the ecotourism organizations must have public reports of residents’ feedback on visitor satisfaction in order to improve the organization’s sustainable ecotourism actions and development. 10. Marketing Database (MD): the ecotourism organizations have to record a complete and accurate marketing database with information regarding the destination and organization, and its products, services, and sustainability claims in order to authentically and respectfully represent the local style and features. 11. Intellectual Property (IP): the ecotourism organizations must have a mechanism for protecting and preserving intellectual property rights.

Thirdly, there are nine sub-criteria in the EC evaluated criteria which are 1. Active Environmental Management (AEM): the ecotourism organizations have to possess a regular system to comprehensively review, evaluate, and improve the practices, with stakeholders’ involvement, in order to administer to the total numbers of tourists and tour activities based on the environmental, economic, socio-cultural, and managerial carrying capacity of the local natural resources [30]. In addition, the system needs to issue a published manual to express the proper responses to issues, such as energy, water, solid waste, harmful substances, etc. 2. Green Usage (GU): the ecotourism organizations have a green usage mechanism for the organization’s purchasing, utilizing, reusing, and recycling in the operational procedures. 3. Solid Waste Management (SWM): the ecotourism organizations are supposed to institute the related guidelines and regulations to construct a system to safely and sustainably measure, monitor, reduce, reuse, and recycle solid waste for the ecotourism stakeholders. 4. Energy Management (EM): the ecotourism organizations have related policies and systems to measure, monitor, publicly report, and reduce energy consumption, including reliance on fossil fuels, energy, and light pollution [31]. The related policies and systems also support the minimization of light pollution for ecotourism stakeholders. 5. Water Management (WM): the ecotourism organizations have to institute a series of policies and establish systems not only to indicate, measure, monitor, and publicly report on water sources but also to reduce water usage and ensure safe and proper wastewater treatment by monitoring drinking and recreational water quality and responding to water quality issues quickly. 6. Noise Pollution and Air Quality Management (NPAQM): the ecotourism organizations have to formulate clear guidelines and regulations to minimize noise pollution for the ecotourism stakeholders. 7. Greenhouse Carbon Emissions Management (GCEM): the ecotourism organizations have to build a complete and record keeping system to measure, monitor, publicly report, minimize, and mitigate their greenhouse carbon emissions from all aspects of their ecotourism activities in order to maintain good air quality for the ecotourism stakeholders. 8. Low-impact Transportation on Environment (LTE): the ecotourism organizations must have a completely green transportation system to advance the usage of low-impact public transportation and active transportation. 9. Wildlife Safeguard (WS): the ecotourism organizations should set up a professional system to ensure compliance with national and international laws and standards for the harvest or capture, consumption, display, and sale of wildlife, including plants and animals, in order to receive contributions for conservation from the ecotourism stakeholders [32].

Fourthly, there are three sub-criteria in the GEEP evaluated criteria which are 1. Nature and Cultural Experiences (NCE): the ecotourism organizations are supposed to supply tourist activities with consideration to the individual enjoying, experiencing, and understanding the nature and culture of the destination. 2. Interpretation Services (IS): the ecotourism organizations have to offer accurate interpretive information at natural and cultural sites. The local information is comprehensive, culturally appropriate, developed with community collaboration, and communicated in languages pertinent to tourists. 3. Interpretation Planning and execution (IPE): the ecotourism organizations have to design a series of plans and manuals for interpretation service and review and regularly improve these plans and manuals [33].

Fifthly, there is one core sub-criteria in the RTBI evaluated criteria which is the Tourist Management (TM): the ecotourism organizations have to plan and design a strong tourism system, developed with the consent of and in collaboration with the affected community, to strictly protect natural and cultural assets and sites and take into account the cumulative impacts of interactions with wildlife, regarding the sale, trade, display, or gifting of local assets and in consideration of historical and archaeological artifacts.

Sixthly, there are four sub-criteria in the LPBS evaluated criteria which are 1. Local Participation and Benefits Sharing Duties (LPBSD): the ecotourism organizations should contribute to the local welfare of the ecotourism stakeholders. 2. Support for Capacity Building of the Local Community (SCBLC): the ecotourism organizations have to create and design various training programs and courses to enhance the local community’s awareness and capability of ecotourism development. 3. Cooperation with Local Community (CLC): the ecotourism organizations have to establish a complete system that stimulates planning and decision-making on an ongoing basis of local community participation and regularly monitors, records, and reflects their opinions. The local community access to natural and cultural sites is also monitored, protected, and, if necessary, rehabilitated or restored. 4. Supports for the Local Enterprises (SLE): the ecotourism organizations have to construct a cultural preservation system, based on the area’s history, culture, and natural attributes, to develop and sell sustainable and valuable local products through fair trade principles [34].

### 2.2. Literature on Analytical Theories

In order to comprehensively evaluate the interplays and interconnections among the three analytical perspectives (ET, ED, and EA), the four essential issues (MI, SI, CI, and EI) of the GSTC, the six assessed dimensions of ecotourism resolution from the WCC, and the twenty-nine assessed indexes of the KES ecotourism evaluations must be analyzed. To comprehensively explore the sustainable determinants of Taiwanese ecotourism with international standards, the SIT behavioral theory was applied to estimate the interactive dependencies and relationships among individuals, organizations, and society. The reason is the SIT was able to be selected to comprehensively assess the in-group favoritism and out-group discrimination as well as the intergroup conflict appearance due to direct group competition from people who have recently become part of the group.

Ref. [35] used the SIT to illustrate the cognitive events that cause individuals to identify their belongingness and group-ships and, at the same time, the individual motivational ideas that positively assist in obtaining this social identity in their belonging groups. The social identity is formed from the individual accepting the group’s spirits and valued denotations after the individual has recognized his/her own belongingness in the group. Theoretically, the five briefest identified categories in the SLT core principles are sorted into the interconnections among individuals (individualism), organizations (organizationism), and society (socializationism) [36]. These briefly identified categories include (1) social approval (SA): the entire society directly responds, cognizes, and further supports and influences each individual attitude, behavior, and action as well as the organizational development [37]; (2) social reinforcement (SR): the majority of persons and organizations aggressively desire to be identified through a series of studying and learning the professional social skills to deal with a bulk of behavioral norms and organizational differences with reference to public acceptance and approval; (3) social exchange (SE): each individual follows the operational conditioning principles which means the interactions among the individuals, organization, and entire society are decided by the behavioral results through the social identity [38]. The interactions will continue if the individual behavioral results and organizational development are positive; the interactions will stop if the behavioral results and organizational development are negative [39]; (4) social categorization (SCA): the individuals are classified by their personality and characteristics into their belonging groups (organizations) and then, the individuals are further defined by their social categorization [40]; and (5) social comparison (SCP): the individuals compare their belonging group (organizations) with other groups (organizations) in terms of the group’s prestige and social standing to obtain higher self-esteem; significantly, in-belonging groups (organizations) will not compare themselves with just any out-groups because the SC must be relative to each situation and condition.

### 2.3. Literature on Statistical Methods

In the current social science research, the FA of quantitative analysis has been the mainstream quantitative methodology for administering large-scale questionnaires by detecting the interplays and dependencies among each evaluated criterion. It is able to comprehensively assay a bulk of complicated research questions and issues, according to its theoretical concept, analytical design and structure, and statistical method. For this reason, this research employed the FA of quantitative analysis to handle the assessments of 247 valid large-scale questionnaires. The entire commonalities among the three analytical perspectives (ET, ED, and EA), the four essential issues (MI, SI, CI, and EI) of the GSTC, the six assessed dimensions of ecotourism resolution from the WCC, and the twenty-nine assessed indexes of the KES ecotourism evaluations were explored in-depth by the FA of quantitative analysis [41]. According to the statistical definition and estimation structure of the FA of quantitative analysis, the dependent variables (directly observed impact-measured factors) were defined as $Y(y_{1},y_{2},\ldots ,y_{k})$ while the independent variables (direct unobserved influenced factors) were defined as $X(x_{1},x_{2},\ldots ,x_{k})$. As for the statistic calculations of FA, the linear combination equation was formed as

(1) $$Y_{k}=W_{k1}X_{1}+W_{k2}X_{2}+\ldots +W_{kk}X_{k}$$

Such that, 1: K numbers of common potential factors were defined from the L numbers of general influenced factors.

2: the M numbers are more than the K numbers.

Then, the weights constants (*W* ($W_{ij}$)) in FA of quantitative analysis could be classified as the evaluated variable loadings of each evaluated criterion and the variable weights of the entire appraised factors under the linear combination Equation (2), based on Equation (1), can be induced as follows:

$$Y_{1}=\lambda_{11}X_{1}+\lambda_{12}X_{2}+\ldots +\lambda_{jk}X_{k}$$

Such that 1: $Y_{ij}=P^{1}X_{ij},X_{ij}=P^{1}Y_{ij}$.

Such that 2: the standardizes intersection of variance is 1 (Max).

If maximization: $X_{k}-u_{k}=\lambda_{k1}f_{1}+\lambda_{k2}f_{2}+\ldots +\lambda_{km}f_{m}+e_{k}$, 

(s.t. ${\left(X-u\right)}_{{-}_{k\times 1}}=\wedge_{m_{k\times m}}f_{m\times 1}+e_{{-}_{k\times 1}}$) the variance-covariance matrix presents as:

(2) $$\sum =\wedge \Psi \wedge^{1}+\Psi_{2}\Psi =diog(\Psi_{1},\Psi_{2},\ldots ,\Psi_{k})\;(s.t.\;\Phi =I_{m*m})$$

Furthermore, the directly observed evaluated factors were defined as the dependent variables (directly observed impact-measured factors), $Y(y_{1},y_{2},\ldots ,y_{k})$, and the directly unobserved evaluated factors were defined as the independent variables (direct unobserved influenced factors) $X(x_{1},x_{2},\ldots ,x_{k})$. Then, the constants of each factor were defined as $W_{ij}$ (representing the factor loading in the FA of quantitative analysis through the weighted measurements of all the influenced factors).

In order to enforce the research reliability and exactness, the ANP of qualitative analysis was further applied for dealing with the expert questionnaires to construct an analytical hierarchy in order to systematically analyze the entire cause and effect among each appraised attitude, evaluated assessed criterion and sub-criterion, and candidate. In view of the theoretical inducement of ANP of qualitative analysis, [42] mentioned the ANP was created from the analytical hierarchy process (AHP) for assaying the more complicated research subjects and problems because the ANP method was able to analyze the interactive two-way assessments in the entire evaluated hierarchy. However, the AHP method was able to measure one-way assessments in the entire evaluated hierarchy for simple research subjects and problems. Therefore, the interactive pairwise compared matrix of ANP of qualitative analysis was formed as the following:

$$A={\left(\begin{array}{c} 1-a_{1j}-a_{1n}\\ a_{i1}-a_{ij}-a_{in}\\ a_{ij}-a_{nj}-a_{1j} \end{array}\right)}_{n\times n}={\left(\begin{array}{c} W_{1}/W_{1}-W_{1}/W_{j}-W_{1}/W_{n}\\ W_{i}/W_{1}-W_{1}/W_{1}-W_{i}/W_{n}\\ W_{n}/W_{1}-W_{n}/W_{1}-W_{n}/W_{n} \end{array}\right)}_{n\times n}$$

In the interactive pairwise compared matrix, the measured weights were defined as $W_{j}$ and the pairwise ratio between each evaluated criterion was displayed as $W_{i}/W_{j}$. Continuously, there were three kinds of statistic assumptions in the interactive pairwise compared matrix to be displayed as

$$a_{ij}=W_{i}/W_{j};\;a_{\max}=1,\;\mathrm{for}\;i=j,\;a_{ij}\times a_{ji}=1$$

Particularly, the related pairwise weights (*W* (*W* = [$W_{1},\ldots ,\ldots W_{j},\ldots ,W_{n}$]) and the local priority vector *w* (eigenvector) were able to be measured through a succession of assessments of the vector quantities method (AW = *nW*) by calculating the inductive principle (AW = $\lambda_{\max}$) in the interactive pairwise compared matrix.

Eventually, the priority vector and maximized eigenvalue of reciprocal determination between each evaluated criterion were computed in the interactive pairwise compared matrix as well. In association with the verification of the interactive pairwise compared matrix, the two-stage algorithm was computed in Equation (3).

(3) $$Rw=\lambda_{\max}w_{i}w_{j}=\sum_{j=1}^{m}(R_{ij}/\sum_{i=1}^{m}R_{ij})/m$$

Importantly, the consistency index (C.I.) was able to be calculated in each interactive pairwise compared matric and then, the consistency ratio (C.R.) was further able to be estimated through the numbers of C.I. and random index (R.I) computed from the estimated table of random index figure in Equation (4)

(4) $$C.I.=(\lambda_{\max}-n)/(n-1);\;C.R.=C.I./R.I.$$

In terms of the verification of ANP of qualitative analysis, the numbers of the C.R. in each pairwise compared matric need to be lower than 0.1 in the evaluated measurements of each pairwise compared matric.

## 3. Results Design

### 3.1. Research Steps

**Figure 2:**
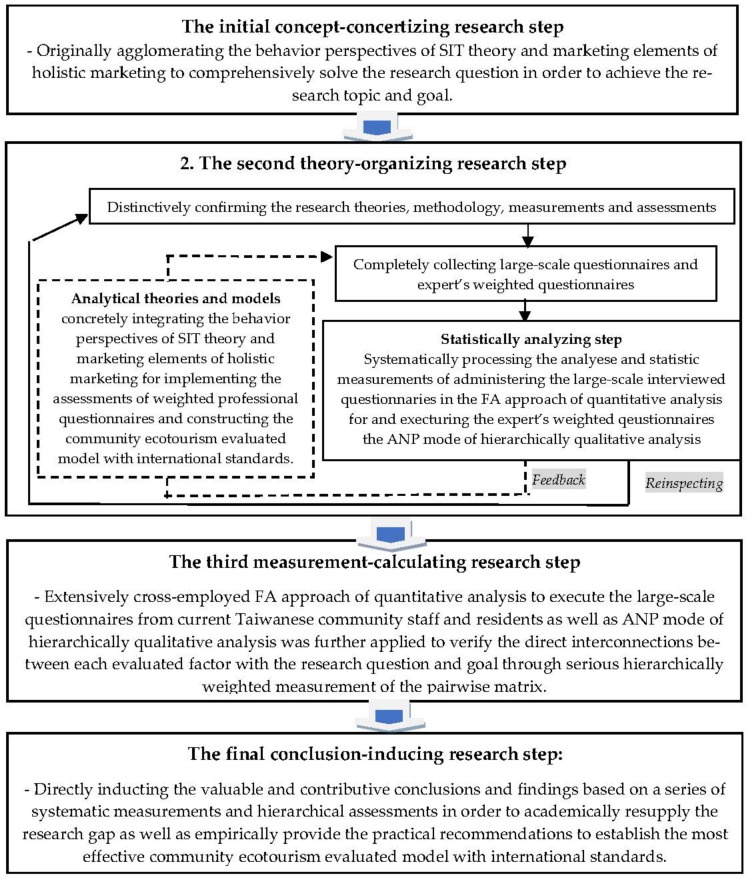
Research design and steps.

This research cross-integrated the behavior perspectives of SIT theory and marketing elements of holistic marketing, not only to compressively identify the determinants of Taiwanese community ecotourism with the international standards, but also to systematically construct the Taiwanese community ecotourism international standard evaluated model. Statistically, not only was the FA of quantitative analysis employed for administering the measurements of large-scale questionnaires but the ANP mode of hierarchically qualitative analysis was also applied for implementing the assessments of the weighted professional questionnaires and constructing the community ecotourism evaluated model with international standards. In detail, there are four essential research steps to be established as referred to in Figure 2:

1. The initial concept-concertizing research step: Originally agglomerating the behavior perspectives of SIT theory and marketing elements of holistic marketing to comprehensively solve the research question in order to achieve the research topic and goal.
2. The second theory-organizing research step: Concretely integrating the behavior perspectives of SIT theory and marketing elements of holistic marketing (as referred to in Figure 1) for implementing the assessments of weighted professional questionnaires and constructing the community ecotourism evaluated model with international standards.
3. The third measurement-calculating research step: Extensively cross-employing the FA approach of quantitative analysis to examine the large-scale questionnaires from current Taiwanese community staff and residents as well as applying the ANP mode of hierarchically qualitative analysis to verify the direct interconnections between each evaluated factor with the research question and goal through a series of hierarchically weighted measurements of the pairwise matrix.
4. The final conclusion-inducing research step: Directly inducting the valuable and contributive conclusions and findings based on a series of systematic measurements and hierarchical assessments in order to academically close the research gap as well as empirically provide the practical recommendations to establish the effective community ecotourism evaluated model with international standards.

The four complete research steps are illustrated step-by-step in Figure 2.

### 3.2. Collection of Data

In order to reach a higher research validity, the sample size of questionnaires has always been a consideration in the FA of quantitative analysis. In light of the sample size of collected questionnaires in the FA of quantitative analysis, ref. [43] addressed the first standpoint that the sample size in FA of quantitative analysis has at least 100; then, ref. [44] demonstrated that the ideal sample size to determine validity should be between 100 to 300 for higher research. Additionally, ref. [45] pointed out that the sample size of collected questionnaires in the FA of quantitative analysis was supposed to be more than five times the evaluated factors in the FA of quantitative analysis for higher research accuracy. Furthermore, ref. [46] explored that the ideal sample size of collected questionnaires was 200 in the FA of quantitative analysis in association with higher research exactness in social science research. Therefore, the original data size had to be designed for a total of 250 Taiwanese ecotourism relative industrialists in view of higher research validity and representativeness and the sampling technique used was the simple random sampling method of probability sampling. In consideration of academic ethical regulations and policies of the National Science and Technology Council in Taiwan and global academic ethical organizations, there are five principles for the free paper and cargo examination in social science research which are (1) all interviewed participants were directly surveyed in the research, (2) no personal information or data regarding interviewed participants’ identification is to be described in research, (3) all interviewed participants provide consent for the use of their accomplished questionnaires, (4) all interviewed participants have to be older than the legal adult age, and (5) the surveyed method does not involve any kind of invasive measures. Therefore, the 247 interviewed participants of the large-scale questionnaires used in this research were older than 18 years of age, higher education students who were older than the Taiwanese legal adults’ age of 18 years, and they all consented to the use of their questionnaires. The surveyed measures are interviews and thus, there were no invasive measures in the interview processes. Additionally, no individual-identified information and data were exposed in this research for the evaluation of the FA approach of quantitative analysis.

Subsequently, ref. [47] addressed that the presented expert’s and professional’s collected questionnaires should be at least over ten percent of surveyed data to ensure the least errors of research validity and reliability due to the date-collection Delphi method. Thus, considering over ten percent of the 250-person surveyed data, there were 20 experts, professionals, and specialists who were in-person interviewed for expertly weighted questionnaires in the ANP of qualitative analysis [48]. These twenty experts, professionals, and specialists contained five scholars who have over ten research experiences in the human behavior of community residents and five specialists who possess over ten working experiences in various community tourism organizations [49]. Furthermore, five professionals have over five empirical work experiences in the community’s operations, and the last five specialists possess over ten working experiences serving in government community related departments [50].

## 4. Results Measurements

### 4.1. FA Measurements of Quantitative Analysis

For the implementation of the FA of quantitative analysis, large-scale weight questionnaires were designed for 250 current Taiwanese tourism-related industrialists. The collection method was an in-person interview approach. The number of valid questionnaires collected was 247 out of the total 250 so the percentage of valid retrieved large-scale questionnaires was 98.8%. These valid 247 surveys cover the northern Taiwanese region (Chilung, Taipei, New Taipei, and Taoyuan cities), middle region (Hsinchu, Miaoli, Taichung, and Changhua cities), southern region (Yunlin, Chiayi, Tainan, and Kaohsiung cities), and eastern region (Yilan, Hualien, and Taitung cities). The descriptive statistic of the FA of quantitative analysis is expressed in Table 1. Significantly, only 8 (3.24%) of the valid 247 interviewees had heard of the ecotourism evaluated indicators and only 39 (15.79%) of the 247 interviewees had heard of sustainable ecotourism with international standards before.

With reference to Equation (1) of the FA approach of quantitative analysis, Table 2 shows that the analytical Kaiser–Meyer–Olkin measure of sampling adequacy was 0.841, which is higher than 0.7, and the significance number of the Kaiser–Meyer–Olkin measure and Barlett test was 0.000, which is smaller than 0.05. Overall, the FA approach of quantitative analysis was suitable for measuring these large-scale questionnaires.

### 4.2. Results of the FA Measurements of Quantitative Analysis

The entire commonalities of each appraised criterion of FA of quantitative analysis are shown in Table 3; the commonality expresses the interplays between each evaluated factor. Specifically, the commonalities of evaluated criteria and sub-criteria are as follows: EFD (criterion) was 0.625, RM (criterion) was 0.623, EC (criterion) was 0.647, GEEP (criterion) was 0.629, RTBI (criterion) was 0.614, LPBS (criterion) was 0.625, RP (sub-criterion) was 0.607, ECBI (sub-criterion) was 0.645, CL (sub-criterion) was 0.683, FI (sub-criterion) was 0.695, SMS (sub-criterion) was 0.753, MU (sub-criterion) was 0.698, M (sub-criterion) was 0.615, SCC (sub-criterion) was 0.728, PR (sub-criterion) was 0.674, CEM (sub-criterion) was 0.697, VS (sub-criterion) was 0.607, MD (sub-criterion) was 0.625, IP (sub-criterion) was 0.704, AEM (sub-criterion) was 0.729, GU (sub-criterion) was 0.614, SWM (sub-criterion) was 0.635, EM (sub-criterion) was 0.667, WM (sub-criterion) was 0.675, NPAQM (sub-criterion) was 0.693, GCEM (sub-criterion) was 0.635, LTE (sub-criterion) was 0.653, WS (sub-criterion) was 0.718, NCE (sub-criterion) was 0.645, IS (sub-criterion) was 0.718, IPE (sub-criterion) was 0.634, TM (sub-criterion) was 0.617, LPBSD (sub-criterion) was 0.702, SCBLC (sub-criterion) was 0.743, CLC (sub-criterion) was 0.721 and SLE (sub-criterion) was 0.703. Significantly, the entire commonalities were higher than 0.6, which indicates higher interconnected interplays and dependences between each evaluated attitude, criterion, and sub-criterion with respect to the research question and goal.

### 4.3. ANP Measurements of Qualitative Analysis

**Figure 3:**
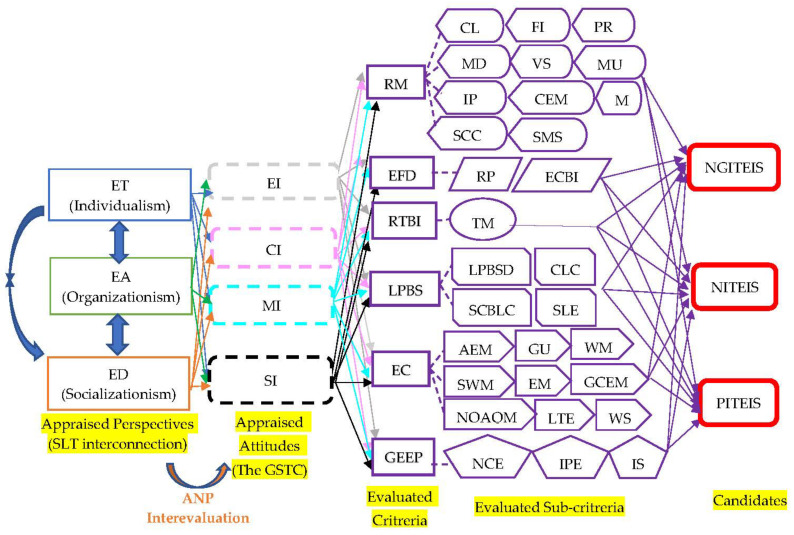
The ANP-evaluated hierarchy.

After administering the FA of quantitative analysis, the evaluated model was established to identify the sustainable determinants of Taiwanese ecotourism with the international standards by verifying the interconnections among the appraised perspectives and attitudes, evaluated criteria and sub-criteria, and the following candidates: Negative Impact on the Taiwanese Ecotourism with the International Standards (NGITEIS), No Impact on the Taiwanese Ecotourism with the International Standards (NITEIS), and Positive Impact on the Taiwanese Ecotourism with the International Standards (PITEIS), as shown in Figure 3.

In association with Figure 3, the pairwise compared mix of ANP hierarchical qualitative analysis among each appraised criterion, sub-criterion, and candidate are shown in Table 4, and mainly, all the C.I. and C.R. numbers in the entire pairwise compared matrix, among appraised attitudes, evaluated criteria and sub-criteria, and candidates, were smaller 0.1 which means the entire pairwise compared matrix consistency of appraised attitudes, evaluated criteria and sub-criteria, and candidates had higher interconnections in an assessed model of ANP of qualitative analysis.

### 4.4. Results of the ANP Measurements of Qualitative Analysis

As a result, Table 5 illustrates the assessed calculated consequences of the entire evaluated pairwise compared matrix of the ANP of qualitative analysis in association with the commonalities of the FA of quantitative analysis in order to achieve higher research reliability, representativeness, validity, and accuracy. Consequently and comprehensively, the highest of the Synthetically Comparative Weighted Numbers (SCWN) was practically located in the PITEIS (0.7226).

## 5. Conclusions and Recommendations

This research employed the SLT in the basic theory of social psychology to comprehensively assay the interplays and interconnections among the three analytical perspectives (ET, ED, and EA), the four essential issues (MI, SI, CI, and EI) of the GSTC, the six assessed dimensions of ecotourism resolution from the WCC, and the twenty-nine assessed indexes of the KES ecotourism evaluations. It was able to comprehensively explore the sustainable determinants of Taiwanese ecotourism with the international standards through the FA of quantitative and qualitative analyses in connection with the highest research validity, reliability, representativeness, and accuracy. After administering a succession of the evaluated measurements, the empirical and valuable conclusions and findings are:

(1) The three analytical perspectives (ET, ED, and EA), the four essential issues (MI, SI, CI, and EI) of the GSTC, the six assessed dimensions of ecotourism resolution from the WCC, and the twenty-nine assessed indexes of the KES ecotourism evaluations positively advance Taiwanese ecotourism with the international standards (PITEEICIS), empirically closing the research gap in the current related Taiwanese ecotourism research fields and effectively providing the political recommendations for the sustainable ecotourism development for the Taiwanese government.
(2) The top five synthetically comparative weights in the PITEEICIS are the sustainable determinants of Taiwanese ecotourism with the international standards and these are the Support for Capacity Building of the Local Community (SCBLC), Cooperation with the Local Community (CLC), Supports for the Local Enterprises (SLE), Local Participation and Benefits Sharing Duties (LPBSD), and the Local Participation and Benefits Sharing (LPBS) as well as Tourist Management and Responsible Tourist Behaviors Inducement (RTBI). Importantly, the majority of ecotourism industrialists and experts still focus on sharing the economic benefits, such as supporting the local community and enterprises, rather than inducing tourist behaviors to stimulate ecotourism participation in order to promote and advance Taiwanese ecotourism with the international standards.
(3) In order to promote Taiwanese ecotourism to the international conventions, the Taiwanese government and organizations in ecotourism should contribute to local welfare and create and design various training programs and courses to enhance the local community’s awareness and capability of ecotourism development in order to establish a complete system that stimulates the ongoing planning and decision making of local community participation and regularly monitors, records, and reflects their opinions, based on the area’s history, culture, and natural attributes, to develop and sell valuable and sustainable local products through the fair trade principles.
(4) In particular, taking the Taiwanese government in line with international practices, the Taiwanese government and organizations in ecotourism need to plan and design a strong tourism system, developed with the consent of and in collaboration with the affected community, to strictly protect natural and cultural assets and sites and take into account the cumulative impacts of interactions with wildlife regarding the sale, trade, display, or gifting of local assets in consideration of historical and archaeological artifacts.

## 6. Discussion

With reference to the sustainable development of ecotourism in Taiwan, the internationalization of ecotourism has been imperative. Hence, Taiwan has to develop and establish a comprehensive sustainable ecotourism institution and system with the international standards. However, to the best of our knowledge, there is not any available research that comprehensively analyzes and develops a complete model to detect the sustainable determinants of Taiwanese ecotourism with international standards. To academically fill this gap, this research employed the SLT and was able to discover the correlations among the four essential issues (Management Issue, MI; Social Issue, SI; Cultural Issue, CI; and Environmental Issue, EI) of the GSTC, the six assessed dimensions of ecotourism resolution from the WCC, and the twenty-nine assessed indexes of the KES ecotourism evaluations in the main analytical hierarchy and concept.

In terms of future directions, evaluated results, and measured consequences, this research was able to explore the sustainable determinants of Taiwanese ecotourism with the international standards in order to create an evaluation mechanism which adopts the international practices and accreditations of the WCC and CSTC.

Specifically, with respect to the research limitations, this research may be restricted to Taiwan; however, this research originally focused on the sustainable determinants of Taiwanese ecotourism with the international standards. In addition, although this research employed the SLT of the basic theory of social psychology to fill the current research gap in the sustainable determinants of Taiwanese ecotourism with the international standards with quantitative and qualitative analyses, there are still multiple decision-making methods and theories to be used in order to detect more key sustainable determinants for completing the Taiwanese ecotourism development and certifications with the international standards.

## Figures and Tables

**Table 1:** Descriptive statistics of the FA approach.

Gender	Male: 148 (59.92%)	Female: 99 (48.08%)		
Geography	Northern Taiwan ^1^: 91 (36.84%)	Middle Taiwan ^2^: 93 (37.65%)	Southern Taiwan ^3^: 51 (20.64%)	Eastern Taiwan ^4^: 12 (4.87%)
Have you heard of ecotourism before?			Yes: 193 (78.13%)	No: 54 (21.87%)
Have you heard of sustainable ecotourism before?			Yes: 8 (3.24%)	No: 239 (96.76%)
Have you heard of sustainable ecotourism with international standards before?			Yes: 39 (15.79%)	No: 208 (84.21%)
Will you respect ecotourism regulations while administering tourism work?			Yes: 202 (81.78%)	No: 45 (18.22%)

^1^: Chilung, Taipei, New Taipei, and Taoyuan cities. ^2^: Hsinchu, Miaoli, Taichung, and Changhua cities. ^3^: Yunlin, Chiayi, Tainan, and Kaohsiung cities. ^4^: Hualien and Taitung counties.

**Table 2:** KMO and Bartlett’s test of FA approach.

Kaiser–Meyer–Olkin Measure of Sampling Adequacy		0.841
Bartlett test of sphericity	Chi-squared test	557.084
	df	171
	Significance	0.000

**Table 3:** The commonality of each assessed criterion in FA the of quantitative analysis.

Appraised Criteria and Candidates	Initial	Extraction
EFD (criterion)	1	0.625
RM (criterion)	1	0.623
EC (criterion)	1	0.647
GEEP (criterion)	1	0.629
RTBI (criterion)	1	0.614
LPBS (criterion)	1	0.625
RP (sub-criterion)	1	0.607
ECBI (sub-criterion)	1	0.645
CL (sub-criterion)	1	0.683
FI (sub-criterion)	1	0.695
SMS (sub-criterion)	1	0.753
MU (sub-criterion)	1	0.698
M (sub-criterion)	1	0.615
SCC (sub-criterion)	1	0.728
PR (sub-criterion)	1	0.674
CEM (sub-criterion)	1	0.697
VS (sub-criterion)	1	0.607
MD (sub-criterion)	1	0.625
IP (sub-criterion)	1	0.704
AEM (sub-criterion)	1	0.729
GU (sub-criterion)	1	0.614
SWM (sub-criterion)	1	0.635
EM (sub-criterion)	1	0.667
WM (sub-criterion)	1	0.675
NPAQM (sub-criterion)	1	0.693
GCEM (sub-criterion)	1	0.635
LTE (sub-criterion)	1	0.653
WS (sub-criterion)	1	0.718
NCE (sub-criterion)	1	0.645
IS (sub-criterion)	1	0.718
IPE (sub-criterion)	1	0.634
TM (sub-criterion)	1	0.617
LPBSD (sub-criterion)	1	0.702
SCBLC (sub-criterion)	1	0.743
CLC (sub-criterion)	1	0.721
SLE (sub-criterion)	1	0.703

Extraction method: Principal component analysis of the FA approach.

**Table 4:** The entire commonalities of each assessed criterion, sub-criterion, and candidate.

Pairwise-Comparison Matrix	C.I.	C.R.
ET	0.0797	0.0885
EA	0.0837	0.0837
ED	0.078	0.0867
EI	0.0426	0.0765
CI	0.0536	0.0781
MI	0.0534	0.0952
SI	0.0458	0.079
RP	0.0443	0.0765
ECBI	0.0453	0.0781
CL	0.0552	0.0952
FI	0.0576	0.0993
SMS	0.0548	0.0944
MU	0.0348	0.06
M	0.057	0.0983
SCC	0.0501	0.0863
PR	0.0536	0.0924
CEM	0.0508	0.0876
VS	0.0568	0.0979
MD	0.0568	0.0979
IP	0.0466	0.0803
AEM	0.0495	0.0854
GU	0.0423	0.0729
SWM	0.0468	0.0807
EM	0.0528	0.091
WM	0.0534	0.0921
NPAQM	0.0544	0.0938
GCEM	0.0432	0.0745
LTE	0.0421	0.0726
WS	0.0505	0.0871
NCE	0.0468	0.0807
IS	0.0435	0.0751
IPE	0.0483	0.0833
TM	0.0381	0.0658
LPBSD	0.0522	0.0899
SCBLC	0.0547	0.0943
CLC	0.0564	0.0972
SLE	0.0514	0.0886

**Table 5:** The evaluated measurements of the ANP hierarchical model.

				NGITEIS		NITEIS		PITEIS	
Criteria	Weights	Sub-Criteria	FA-Communalities	Weight	Evaluated Score	Weight	Evaluated Score	Weight	Evaluated Score
EFD	0.0577	RP	0.607	0.06	0.0021	0.2112	0.0074	0.7288	0.0255
		ECBI	0.645	0.0605	0.0022	0.2077	0.0077	0.7318	0.0272
RM	0.0277	CL	0.683	0.0569	0.0011	0.2068	0.0039	0.7364	0.0139
		FI	0.695	0.0615	0.0012	0.2203	0.0042	0.7182	0.0138
		SMS	0.753	0.0623	0.0013	0.2239	0.0047	0.7138	0.0149
		MU	0.698	0.0655	0.0013	0.2297	0.0044	0.7048	0.0136
		M	0.615	0.0599	0.001	0.2235	0.0038	0.7167	0.0122
		SCC	0.728	0.0617	0.0012	0.2239	0.0045	0.7144	0.0144
		PR	0.674	0.0551	0.001	0.2092	0.0039	0.7357	0.0137
		CEM	0.697	0.0571	0.0011	0.2057	0.004	0.7373	0.0142
		VS	0.607	0.0624	0.001	0.2204	0.0037	0.7172	0.0121
		MD	0.625	0.0593	0.001	0.2253	0.0039	0.7154	0.0124
		IP	0.704	0.0573	0.0011	0.2151	0.0042	0.7276	0.0142
EC	0.0773	AEM	0.729	0.0569	0.0032	0.2171	0.0122	0.7259	0.1276
		GU	0.614	0.056	0.0027	0.2125	0.0101	0.7315	0.0347
		SWM	0.635	0.0582	0.0029	0.215	0.0106	0.7268	0.0357
		EM	0.667	0.0608	0.0031	0.2206	0.0114	0.7185	0.0371
		WM	0.675	0.0605	0.0032	0.2235	0.0117	0.716	0.0374
		NPAQM	0.693	0.0615	0.0033	0.2342	0.0126	0.7043	0.0378
		GCEM	0.635	0.0559	0.0027	0.2257	0.0111	0.7184	0.0353
		LTE	0.653	0.0561	0.0028	0.2248	0.0114	0.7191	0.0363
		WS	0.718	0.059	0.0033	0.2299	0.0128	0.7111	0.0395
GEEP	0.241	NCE	0.645	0.0597	0.0093	0.2272	0.0353	0.7131	0.1109
		IS	0.718	0.0601	0.0104	0.2267	0.0392	0.7132	0.1234
		IPE	0.634	0.0592	0.0091	0.2302	0.0352	0.7105	0.1086
RTBI	0.6539	TM	0.617	0.058	0.0234	0.2234	0.0901	0.7186	0.2899
LPBS	0.7390	LPBSD	0.702	0.06	0.0311	0.2249	0.1167	0.715	0.3709
		SCBLC	0.743	0.0624	0.0343	0.2241	0.1231	0.7135	0.3917
		CLC	0.721	0.0621	0.0331	0.2252	0.12	0.7126	0.3797
		SLE	0.703	0.06	0.0312	0.2208	0.1147	0.7192	0.3736
SCWN					0.0588		0.2185		0.7226

## Abbreviations

WCC	World Conservation Congress
USD	United States Dollar
COVID-19	Coronavirus Disease-2019
IUCN	International Union for the Conservation of Nature
ED	Eco-friendly Development
RM	Responsible Management
EC	Environmental Conservation
GEEP	Genuine Ecotourism Experiences Provision
RTBI	Responsible Tourist Behavior Inducement
LPBS	Local Participation and Benefits Sharing
UNSD	United Nations Statistics Division
KES	Korean Ecotourism Standards
RP	Resources Protection
ECBI	Eco-friendly and Cultural-friendly Buildings and Infrastructures
CL	Compliance with Laws
FI	Financial Insurance
SMS	Sustainable Management Strategies
MU	Management Units
M	Monitoring
SCC	Strategy for Climate Change
PR	Planning Regulations
CEM	Crisis and Emergency Management
MD	Marketing Database
IP	Intellectual Property
AEM	Active Environmental Management
GU	Green Usage
SWM	Solid Waste Management
EM	Energy Management
WM	Water Management
NPAQM	Noise Pollution and Air Quality Management
GCEM	Greenhouse Carbon Emissions Management
LTE	Low-impact Transportation on the Environment
WS	Wildlife Safeguard
NCE	Nature and Cultural Experiences
IS	Interpretation Services
IPE	Interpretation Planning and Execution
TM	Tourist Management
LPBSD	Local Participation and Benefits Sharing Duties
SCBLC	Support for Capacity Building of the Local Community
CLC	Cooperation with the Local Community
SLE	Supports for the Local Enterprises
TEA	Taiwan Ecotourism Association
SLT	Social Learning Theory
FA	Factor Analysis
ANP	Analytical Network Process
